# Ultraviolet Photodissociation of the *N*,*N*-Dimethylformamide Cation

**DOI:** 10.1021/acs.jpca.4c06227

**Published:** 2024-12-03

**Authors:** Dennis Milešević, Alexander Butler, P. A. Robertson, Claire Vallance

**Affiliations:** Department of Chemistry, Chemistry Research Laboratory, University of Oxford, 12 Mansfield Rd, Oxford OX1 3TA, U.K.

## Abstract

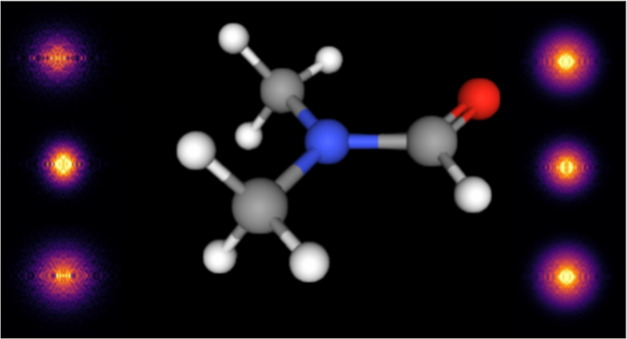

*N*,*N*-Dimethylformamide (DMF) provides a useful small-molecule model for studying features of the peptide bond that forms the backbone of proteins. We report results from a comprehensive multimass velocity-map imaging study into the ultraviolet (UV) photolysis of the *N*,*N*-dimethylformamide cation (DMF^+^) at wavelengths of 225, 245, and 280 nm. Electronic structure calculations on DMF and DMF^+^ were employed to help interpret the experimental results. DMF^+^ ions are generated by 118 nm single-photon ionization of neutral DMF. Subsequent UV photolysis is found to lead to selective cleavage of the N–CO amide bond. This yields HCO + NC_2_H_6_^+^ as major products, with virtually all of the excess energy released into internal modes of the fragments. The data also indicate a small branching ratio into the HCO^+^ + NC_2_H_6_ product pair, which can be accessed from the 3^2^A′ electronic state of DMF^+^. N–CO bond dissociation can also be accompanied by simultaneous intramolecular hydrogen transfer from the oxygen to the nitrogen end of the amide bond, in which case NCH_4_^+^ can be formed efficiently at all three wavelengths. The primary NC_2_H_6_^+^ product is relatively long-lived, but the high degree of internal excitation often results in secondary fragmentation via a variety of pathways to form CH_3_^+^, NH_4_^+^, NCH_2_^+^, and NC_2_H_4_^+^, with secondary dissociation more likely at higher photon energies. The isotropic velocity-map images recorded for the various fragments attest to the long lifetime of NC_2_H_6_^+^ and also imply that dissociation most probably occurs from the same set of electronic states at all wavelengths studied; these are thought to be the 1^2^A′ ground state and 2^2^A′ first excited state of the DMF^+^ cation.

## 1 Introduction

Mass spectrometric methods are frequently employed to investigate the fragmentation of peptide cations in the gas phase.^1−3^ Fragmentation processes are typically described using the mobile proton model:^4−8^ long-lived peptide cations are known to undergo facile intramolecular hydrogen atom transfer, resulting in various “charge-directed” bond-breaking mechanisms, in which bond cleavage occurs close to the location of the charge.^9,10^

The amide bond in *N*,*N*-dimethylformamide (DMF) offers a useful model for investigating peptide bond fragmentation dynamics.^11−22^ We have previously presented a pair of comprehensive studies into the photodissociation of neutral DMF^17,22^ at a number of wavelengths in the ultraviolet (UV) region. The present study describes an investigation into the photodissociation dynamics of the cation at several wavelengths in the UV region.

Previous investigations of the cation have focused on the dissociation dynamics following electron ionization. In an electron ionization study on isotopically labeled DMF, Li et al.^23^ provided an unambiguous product ion assignment and reported appearance energies for the various observed product ions. The authors also proposed a number of fragmentation pathways to explain the observed fragment ions. One channel involves cleavage of the N–CO amide bond, which may also involve an intramolecular hydrogen shift from the carbonyl end of the bond to the nitrogen atom, as initially proposed by Loudon and Webb.^24^ We note that such shifts are not observed in dissociation of neutral DMF. Another important channel identified by Li et al. is cleavage of an N–CH_3_ bond, which also occurs in concert with a similar hydrogen transfer. A fragment not observed in dissociation of neutral DMF, but formed in abundance following ionization, is NCH_4_^+^. The dissociation pathway leading to this product requires cleavage of both an N–CH_3_ bond and the N–CO bond, together with hydrogen transfer to the nitrogen atom. Thus, intramolecular hydrogen transfer appears to be a characteristic feature of the dissociation dynamics of the DMF^+^ cation.

In contrast to the electron ionization studies mentioned above, the photoinduced fragmentation dynamics of the DMF^+^ cation have not been studied previously to our knowledge. In the following, we report the results of a multimass velocity-map imaging (VMI) study into the photodissociation dynamics of DMF^+^ at photolysis wavelengths of 225, 245, and 280 nm.

## 2 Methods

### 2.1 Experiments

The VMI spectrometer used in the present work is the same as that employed in our previous study on neutral DMF^22^ and consists of differentially pumped source, interaction, and time-of-flight (ToF) regions maintained at a base pressure of ∼10^–7^ mbar. Helium (BOC, 99.9%) at a pressure of ≈1 bar was bubbled through liquid DMF (Sigma-Aldrich, 99.8%) to generate an ≈0.5% mixture of DMF in helium. Within the source chamber, the gas mixture underwent a supersonic expansion through a pulsed solenoid valve (Parker Hannifin, Series 9, 10 Hz), and the resulting molecular beam was collimated by a 1.5 mm skimmer, separating the source and interaction chambers before passing through the repeller plate of a three-lens VMI ion optics assembly into the interaction region. Here the molecular beam was intersected at right angles by a vacuum ultraviolet (VUV) ionizing laser beam and an UV photolysis laser beam.

The VUV laser beam used to ionize the sample molecules was generated by frequency tripling the third harmonic of an Nd:YAG laser (Continuum Surelite I, pulse length ∼7 ns) in a phase-matched mixture^25^ of 27 mbar xenon (BOC, 99.9%) and 298 mbar argon (BOC, 99.9%).^26−28^ A Glan-Taylor polarizer and half wave plate were used to polarize the VUV light vertically in the image plane, perpendicular to the ToF axis.

Following a short time delay of approximately 15 ns, the molecular beam was intersected by the few mJ, ∼7 ns UV photolysis laser beam from a tunable, pulsed, frequency-doubled dye laser (Sirah Cobra Stretch), pumped by the third harmonic of an Nd:YAG laser (Continuum Surelite II). The photolysis beam was also linearly polarized in the imaging plane, perpendicular to the ToF axis. Photolysis wavelengths of 225, 245, and 280 nm were employed in this study.

The VMI ion lens consists of a repeller, extractor, and ground electrodes. The repeller plate has a 4 mm diameter hole in the center to admit the molecular beam to the interaction region, and the extractor and ground electrodes have 24 mm holes in the center to transmit the expanding product scattering distributions to the ToF tube. The ratio of potentials applied to the extractor and repeller plates is tuned to achieve velocity-mapping conditions,^29,30^ mapping the three-dimensional scattering distribution of the nascent photofragments via a 56 cm flight tube onto a two-dimensional position-sensitive ion detector.

The ion detector (Photonis) comprises a pair of chevron-mounted microchannel plates coupled to a P47 phosphor screen. Each incoming ion generates an optical signal on the phosphor, which is captured by a second-generation Pixel Imaging Mass Spectrometry (PImMS2) camera.^31−33^ The camera records an (*x*, *y*, *t*) data point for each detected ion, and after centroiding of the individual ion hits (see below) the resulting data set can be integrated over the (*x*, *y*) coordinates to obtain the product ToF spectrum or over the relevant arrival time ranges to generate velocity-map images for each photofragment.

Interaction of neutral DMF with either laser in isolation can lead to the generation of ions; hence, a background subtraction is required in order to isolate the two-laser signal of interest. To achieve this, in addition to the data sets obtained using both the ionization and photolysis laser, single-laser data sets were also acquired. The one-color data sets were subtracted from the two-color data in order to obtain the true two-color signal.

### 2.2 Data Processing

Each ion signal generally excites multiple pixels and time bins within the PImMS2 sensor, leading to some blurring of the images. This can be corrected by centroiding the data set to reduce each ion signal to a single point in the (*x*, *y*, *t*) space.

The velocity-map images recorded in our experiment are two-dimensional (2D) projections of the full three-dimensional (3D) scattering distributions for the relevant fragment ions. The 3D distributions are cylindrically symmetric about the laser polarization vectors, with the result that once the velocity-map images of interest have been extracted from the data set, an inverse Abel transform^34^ can be used to reconstruct the full 3D scattering distribution and to extract the central slice, which contains all of the information needed to determine the product angular and kinetic energy distributions. The Abel inversion was performed using the BaseX software package.^35^ The radial and angular distributions are then extracted from the inverted images by integration over the angular and radical coordinates, respectively, and the radial distributions are converted into kinetic energy distributions using a calibration determined through VMI measurements of the (known) velocity distributions of Cl atoms generated in the 355 nm photolysis of Cl_2_.

### 2.3 Electronic Structure Calculations

The ground-state geometries of neutral DMF and DMF^+^ were optimized at the UMP2/aug-cc-pVTZ level using the Gaussian09^36^ software package. Vertical excitation energies to the first ten ionic states were calculated at the two equilibrium geometries using UB3LYP/aug-cc-pVTZ. Starting from the optimized geometry of neutral DMF, rigid (i.e., all other degrees of freedom fixed) potential energy curves along the N–CO and both N–CH_3_ stretch coordinates were calculated using CASSCF(11,12)/ANO-R2(6-311G**) within the MOLCAS^37^ software package. These reveal the product pair formed upon cleavage of the selected bond. Accurate energies for each product channel have been calculated previously by Li et al.^23^ using the W1BD procedure within Gaussian09 and are also used in interpreting our data.

## 3 Results and Discussion

### 3.1 Electronic Structure Calculations

**Figure 1:**
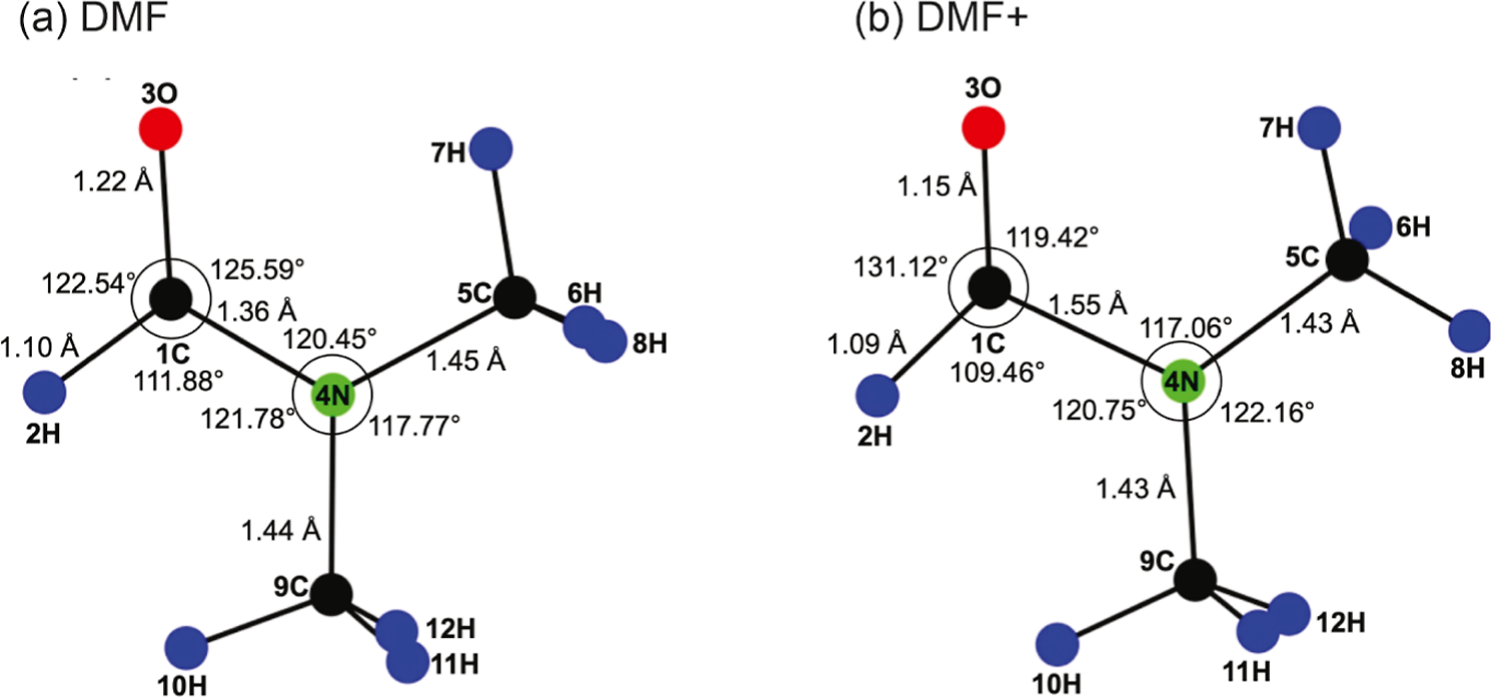
Equilibrium geometries of (a) DMF and (b) DMF^+^ optimized in UMP2/aug-cc-pVTZ calculations. Carbon atoms are shown in black, oxygen atoms in red, nitrogen atoms in green, and hydrogen atoms in blue. The main bond lengths and angles are highlighted in the figure. A full list of internal coordinates is provided in the Supporting Information.

The equilibrium geometries identified through the electronic structure calculations for the ground states of ionized and neutral DMF are shown in Figure 1, with the optimized internal coordinates for each species given in the Supporting Information. With the exception of the methyl-group hydrogen atoms, neutral DMF has a planar structure due to the partial double bond character of the N–CO bond associated with conjugation of the nitrogen lone pair into the amide bond. The 10.49 eV photon energy of the 118 nm ionization laser is sufficient to ionize an electron from either of the two highest lying molecular orbitals. The highest occupied molecular orbital (HOMO) corresponds predominantly to the nitrogen lone pair. Upon ionization from the HOMO, the double bond character of the N–CO amide bond vanishes. The optimized structure of the ion is also almost planar (again excepting the methyl hydrogen atoms), in agreement with previous calculations by other authors.^38^

The N–CO and C=O bond lengths both change significantly upon ionization. The N–CO bond elongates from 1.36 to 1.55 Å, consistent with loss of the partial double bond character, while the C=O equilibrium bond length reduces by a more modest amount, from 1.22 to 1.15 Å. The large change in the N–CO bond length is likely to be significant in determining the dominant fragmentation pathways in the ion.

**Figure 2:**
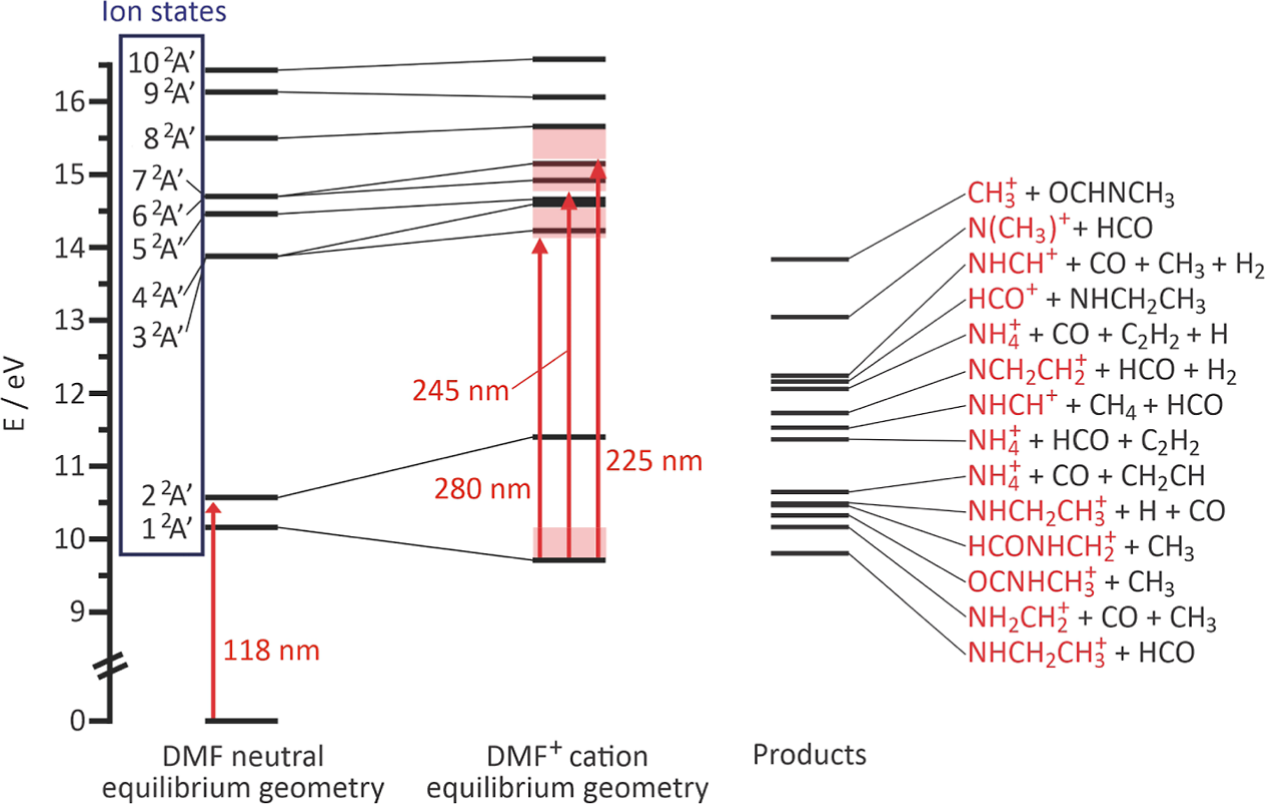
Excitation energies to the first ten electronic states of DMF^+^ at the ground-state geometries of neutral DMF and of the DMF^+^ cation. The red arrows indicate the photoexcitation for ionization (118 nm) and photolysis (225, 245, and 280 nm), and the red-shaded regions show the range of energies that may be accessed depending on the extent of vibrational relaxation of the nascent ions. On the right are the appearance energies for various product channels taken from Li et al.^23^

An energy level diagram showing the calculated energies of the first few electronic states of the DMF cation at the equilibrium geometries of the neutral and cation, together with the energies of the various dissociation products taken from Li et al.,^23^ is shown in Figure 2. Vertical excitation energies from the ground state of neutral DMF to the first ten ionic states are shown in Table 1, together with the products formed from each of the first six states upon cleavage of the N–CO or either of the N–CH_3_ bonds. The product pair assignments were determined from the potential energy curves shown in the Supporting Information.

**Table 1:** Vertical Excitation Energies (*V*_E_) from the Electronic Ground State of Neutral DMF to the Ionic Ground State and Excited States; the Geometries and Energy of the Neutral and Ionic Ground State were Optimized/Calculated with a UMP2/aug-cc-pVTZ Level of Theory; the Vertical Excitation Energies of the Excited States were Calculated Using the UB3LYP/aug-cc-pVTZ Level of Theory; for the First Six Ionic States, the Resulting Product Pair Upon Cleavage of Either the N–CO Bond or Either of the N–CH_3_ Bonds is Shown

state	*V*_E_/eV	N–CO	N–CH_3_
1^2^A′ (ground state)	10.16	HCO + NC_2_H_6_^+^	HCONCH_3_^+^ + CH_3_
2^2^A′	10.57	HCO + NC_2_H_6_^+^	HCONCH_3_^+^ + CH_3_
3^2^A′	13.88	HCO^+^ + NC_2_H_6_	HCONCH_3_^+^ + CH_3_
4^2^A′	13.88	HCO + NC_2_H_6_^+^	HCONCH_3_ + CH_3_^+^
5^2^A′	14.46	HCO + NC_2_H_6_^+^	HCONCH_3_^+^ + CH_3_
6^2^A′	14.70	HCO + NC_2_H_6_^+^	HCONCH_3_^+^ + CH_3_
7^2^A′	14.70		
8^2^A′	15.50		
9^2^A′	16.13		
10^2^A′	16.43		

Experimentally determined vertical ionization potentials for DMF lie in the range from 9.25 to 9.45 eV,^39−42^ somewhat lower than the 10.16 eV predicted by our UMP2/aug-cc-pVTZ calculation. According to our calculated state energies, ionization at 118 nm (10.49 eV) populates almost solely the 1^2^A′ ground state of the ion, though we note that the 2^2^A′ first excited state is also likely to be accessible given our overestimation of the ionization energy. We can therefore be reasonably confident that in our experiments we are photolyzing DMF cations predominantly from their electronic ground state, perhaps with some contribution from the first excited state. We note that relaxation of the nascent ions to the equilibrium geometry of the ionic ground state is likely to generate a modest amount of vibrational excitation prior to the absorption of the UV photon. Without spectroscopic probing of the nascent ions it is not possible to quantify the DMF^+^ internal state distribution prior to UV absorption, but we can place upper and lower bounds on the final energy of the ions following photon absorption and therefore on the accessible electronic states of the ion. These bounds are indicated by the red-shaded rectangles in Figure 2. If we assume rapid relaxation of the ions to the vibrational ground state, then (as shown by the vertical arrows in Figure 2) the highest lying accessible states of the ion at photolysis wavelengths of 280, 245, and 225 nm are the 2^2^A′, 5^2^A′, and 7^2^A′ states, respectively. At the other extreme, if we assume no relaxation, then the total available energy from the two absorbed photons (VUV + UV) becomes 14.93, 15.56, and 16.01 eV for 280, 245, and 225 nm excitation, and the highest accessible states are 4^2^A′, 6^2^A′, and 7^2^A′ at the three excitation energies, respectively. Our conclusions do not change materially between the two limiting scenarios outlined above.

### 3.2 Experimental Data

**Figure 3:**
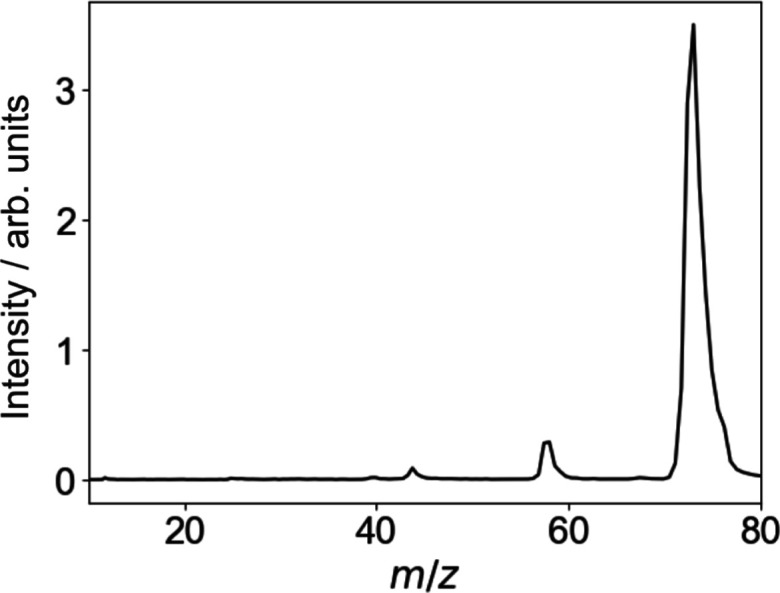
ToF spectrum for the products formed following 118 nm ionization of DMF.

We consider first the product ion ToF mass spectra recorded in the one-laser experiments. We do not see a significant signal when DMF is irradiated only by the UV photolysis laser. However, the ToF mass spectrum observed when neutral DMF is irradiated with 118 nm VUV radiation only is shown in Figure 3. By far, the dominant signal is the DMF^+^ parent ion peak, appearing at *m*/*z* 73. We also observe small fragment ion peaks at *m*/*z* 44 and 58, which we assign to NC_2_H_6_^+^ and HCONCH_3_^+^. The ground state of the DMF^+^ cation correlates to both of these products via cleavage of the N–CO and N–CH_3_ bonds, respectively. The *m*/*z* 44 product has two possible structures: , with an appearance potential^23^ of 13.03 eV, and NHCH_2_CH_3_^+^, with an appearance energy of 10.48 eV when partnered by CO + H or 9.80 eV when partnered by HCO. Given the 10.48 eV photon energy of a 118 nm photon, the latter of these options seems most likely, with the first ruled out on energetic grounds.

The *m*/*z* 58 product of methyl loss from the parent DMF^+^ cation also has several possible structures, with predicted appearance energies^23^ of 13.95, 10.46, and 10.32 eV for the HCONCH_3_^+^, HCONHCH_2_^+^, and OCNHCH_3_^+^ structures, respectively. Again, we can rule out the first of these possibilities on energetic grounds. However, either or both of the latter two structures are compatible with our experimental observations.

The observed ToF spectrum at 118 nm is therefore consistent with the formation of ground-state DMF cations, in line with the vertical excitation energies shown in Table 1 and discussed in Section 3.1.

**Figure 4:**
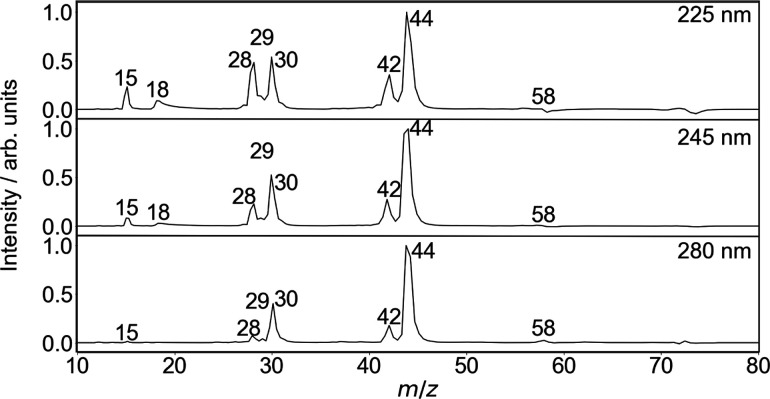
Background-subtracted ToF mass spectra for the photolysis products of DMF^+^ at wavelengths of 225 nm (top panel), 245 nm (center panel), and 280 nm (bottom panel).

ToF mass spectra for the products formed following irradiation of the DMF cations with UV light at wavelengths of 225, 245, and 280 nm are shown in Figure 4, with integrated peak intensities for the observed fragments given in Table 2. The single-laser signals have been subtracted from the two-laser signals in the ToF spectra to leave the true two-color signal. We note that for ions that are only formed in the 118 nm ionization step, in this case the parent ion at *m*/*z* 73 and the HCONCH_3_^+^ ion at *m*/*z* 58, this sometimes leads to slightly negative signals in the difference mass spectra due to depletion of these ions by the photolysis laser in the two-laser experiments.

**Table 2:** Probability (Expressed as a Percentage) of Forming Each Fragment Ion Following Photolysis of DMF^+^ at 225, 245, and 280 nm

ion	*m*/*z*	225 nm	245 nm	280 nm
CH_3_^+^	15	4.6	2.3	<1
NH_4_^+^	18	6.3	2.9	<1
NCH_2_^+^	28	16	8.2	3.5
HCO^+^	29	1.4	1.4	<1
NCH_4_^+^	30	16	20	19
NC_2_H_4_^+^	42	17	12	9.2
NC_2_H_6_^+^	44	39	54	65

**Figure 5:**
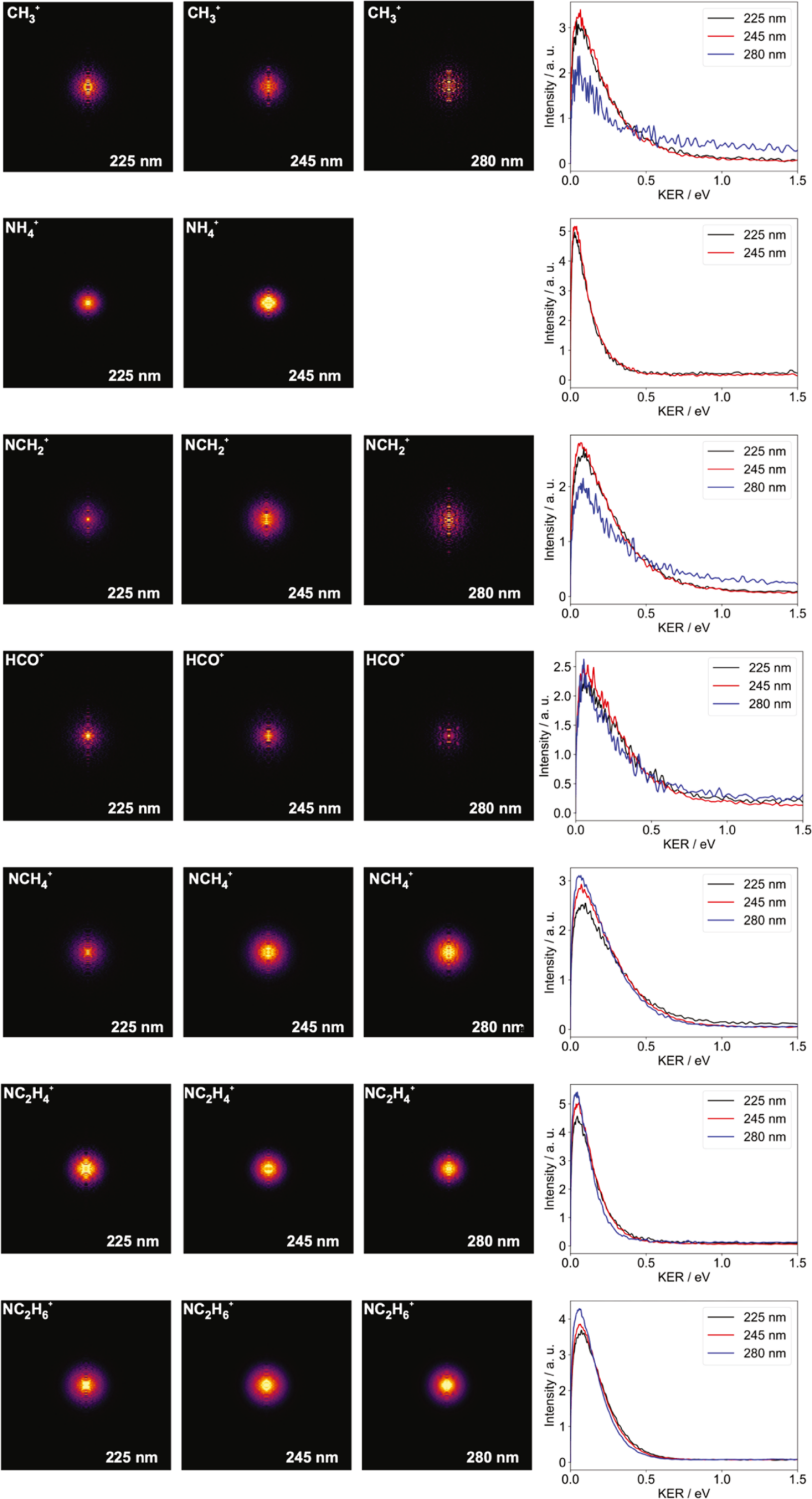
Abel-inverted velocity-map images for the various ionic products formed following photolysis of DMF^+^ at 225, 245, and 280 nm, together with the corresponding kinetic energy distributions (normalized to unit area under the curve) for each ion.

Velocity-map images for the ionic products formed at the three photolysis wavelengths employed are shown in Figure 5, along with the corresponding (normalized) kinetic energy distributions for each fragment ion.

Before considering the various dissociation channels in more detail, we note that the velocity-map images for all detected products are very similar to each other for the three different photolysis wavelengths studied, implying that dissociation most probably occurs from the same electronic state of the ion at all three wavelengths. Based on the vertical excitation energies reported in Table 1, the DMF^+^ cation is excited to the 2^2^A′ state at 280 nm. Dissociation can occur either on this state or on the 1^2^A′ state following internal conversion. Even though excitation at 245 and 225 nm can lead to excitation up to the 7^2^A′ state and 8^2^A′ state, respectively, our results suggest that population transfer occurs rapidly down to the 1^2^A′ and 2^2^A′ states on a time scale faster than the relatively slow dissociation. Dissociation occurring from the ionic ground and first excited states over the photolysis wavelength range studied is consistent with the lack of wavelength dependence observed in any of the velocity-map images.

We now move on to consider first the primary and then the secondary dissociation channels of DMF^+^ following the absorption of a UV photon.

### 3.3 Primary Dissociation Channels

#### 3.3.1 N–CO Amide Bond Cleavage

We focus initially on the primary dissociation channels, involving cleavage of the N–CO or N–CH_3_ bonds. At all three wavelengths studied, the most intense signal appears at *m*/*z* 44, corresponding to the NC_2_H_6_^+^ ion. This ion is the product of a straightforward cleavage of the N–CO amide bond to form HCO + NC_2_H_6_^+^. As noted previously when discussing the ToF spectrum recorded at 11 mi nm, while one might predict the NC_2_H_6_^+^ ion to have the structure , this structure is known to isomerize into the more stable H_2_C–NH–CH_3_^+^ form.^43^ Considering the appearance energies reported for this ion by Li et al.,^23^ we find that experiment and theory are only in agreement if it is assumed that the product is H_2_C–NH–CH_3_^+^, with the  structure predicted to yield an appearance potential several eV higher in energy. The low appearance potential also implies that the rearrangement occurs in concert with the amide bond cleavage rather than occurring in a separate step following formation of the less stable  ion.

From Table 1 we see that the HCO + NC_2_H_6_^+^ products are in principle accessible from many different low-lying electronic states of the ion following cleavage of the amide bond. In contrast, only the 3^2^A′ state yields the charge on the carbon following amide bond cleavage, forming HCO^+^ + NC_2_H_6_. This is in line with the very low HCO^+^ signals observed at *m*/*z* 29 at all three photolysis wavelengths.

Perhaps surprisingly, the kinetic energy distributions for the NC_2_H_6_^+^ fragment ions formed at the three photolysis energies studies are almost identical, extending out only to very slightly higher energies as the photolysis wavelength is reduced from 280 to 225 nm. The KER distributions peak at around 0.05 eV and extend to about 0.75 eV. Rather than being released into product translation, the vast majority of the excess energy is clearly released into the internal excitation of the nascent ion. This is consistent with the increase in the intensity of low-mass peaks in the ToF spectra as the photolysis wavelength is reduced, indicative of increased secondary fragmentation as the internal energy of the primary products increases.

#### 3.3.2 N–CH_3_ Bond Cleavage

We might also expect to see products of N–CH_3_ bond fission following UV excitation of the DMF^+^ cation. According to Table 1, all low-lying states apart from the 4^2^A′ state lead to the HCONCH_3_^+^ + CH_3_ product pair; however, we see virtually no signal at *m*/*z* 58 from HCONCH_3_^+^ at any of the three photolysis energies studied. The 4^2^A′ state correlates to HCONCH_3_ + CH_3_^+^ products on cleavage of an N–CH_3_ bond and is energetically accessible at the 225 and 245 nm photolysis wavelengths. We do observe a CH_3_^+^ ion signal at *m*/*z* 15, which increases in intensity as the photolysis photon energy increases. However, while N–CH_3_ bond fission on the 4^2^A′ state cannot be ruled out, it seems unlikely that N–CH_3_ bond breaking occurs solely on this state and not on lower lying states. It is much more likely that the observed CH_3_^+^ products arise from secondary dissociation of NC_2_H_6_^+^ formed via amide bond fission. We can conclude from the discussion so far that amide bond fission far outpaces methyl loss as a primary photodissociation channel in the DMF^+^ cation.

### 3.4 Secondary Dissociation Channels

Having already identified production of CH_3_^+^ as resulting from secondary dissociation of NC_2_H_6_^+^, we now move on to consider secondary dissociation channels more broadly. From the relative peak intensities given in Table 2, we see that the intensity of the NC_2_H_6_^+^ signal decreases with increasing photon energy, while the signal intensities for the smaller fragment ions CH_3_^+^ (*m*/*z* 15), NH_4_^+^ (*m*/*z* 18), NCH_2_^+^ (*m*/*z* 28), and NC_2_H_4_^+^ (*m*/*z* 42) all increase. This implies that these smaller ions are secondary dissociation products of the primary NC_2_H_6_^+^ ion formed via cleavage of the amide bond, either from the initially formed  or from its more stable isomer H_2_C–NH–CH_3_^+^.

We note that with the exception of CH_3_^+^, the formation of these secondary ions requires significant structural rearrangement of the NC_2_H_6_^+^ ion and is therefore likely to occur over relatively long time scales. An extreme example is found in the case of the NH_4_^+^ ion, for which we see a long exponentially decaying tail on the ToF signal. This is characteristic of the so-called “metastable decay”, in which long-lived parent ions dissociate to form the detected daughter ions outside of the interaction region as they traverse the flight tube to the detector. Similar behavior was observed in our earlier experiments on neutral DMF^17,22^ following ionization of the primary (neutral) HCO and NC_2_H_6_ products for detection. The time scale for the metastable decay leading to production of the observed NH_4_^+^ ions can be estimated to be between ∼100 ns and ∼5 μs, the range of times during which the parent NC_2_H_6_^+^ ion traverses the flight tube to the detector after leaving the interaction region. This is consistent with previous work by Levsen and McLafferty,^43^ which concluded that the nascent NC_2_H_6_^+^ ions have a lifetime of tens of microseconds or more.

The velocity-map images recorded for the secondary dissociation products are very similar both to each other and to those for the NC_2_H_6_^+^ primary products, implying that very little energy is released into translation during the secondary dissociation step. Coupled with the lack of any angular anisotropy in the images, implying molecular rotation prior to dissociation, this is consistent with a lifetime long enough to enable statistical redistribution of energy among all of the available internal modes of the primary NC_2_H_6_^+^ product until a given bond becomes sufficiently energized to break. In common with the behavior observed earlier for the NC_2_H_6_^+^ primary fragmentation product, for the most part the kinetic energy distributions for the secondary dissociation products change only very slightly upon increasing the photon energy (note that the apparent high energy tails on the kinetic energy distributions determined for CH_3_^+^ and NCH_2_^+^ at a photolysis wavelength of 280 nm arise as an artifact from the Abel inversion process; the very low signal levels in these images leads results in a significant amount of centerline noise on the inverted images).

The NCH_4_^+^ ion can be formed via two different three-body dissociation pathways, one of which involves a hydrogen atom transfer from one of the methyl groups to the nitrogen and the other involves a hydrogen transfer across the dissociating amide bond from the carbon to the nitrogen.
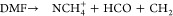
1
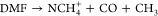
2Interestingly, the channel involving hydrogen atom transfer across the amide bond is significantly lower in energy and accessible at all three photolysis wavelengths studied, while the alternative pathways to NCH_4_^+^ is only energetically accessible at the 225 nm photolysis wavelength. The latter pathway lies considerably higher in energy due to the lower stability of CH_2_ relative to that of CH_3_. The NCH_4_^+^ ion is highly abundant at all three photolysis wavelengths; perhaps surprisingly, its abundance reduces somewhat at the highest photolysis energy, despite a second formation channel becoming energetically accessible. Given the concomitant increase in the yield of NCH_2_^+^, we hypothesize that a higher degree of internal excitation in NCH_4_^+^ formed at 225 nm leads to an enhanced probability of H_2_ loss to form NCH_4_^+^. This is consistent with the appearance energies for the various products published previously by Li et al.^23^

Some further insight into the observed secondary dissociation channels is provided by performing a more detailed comparison with the previously mentioned work of Levsen and McLafferty,^43^ which reports results from a collisional dissociation study on NC_2_H_6_^+^ and deuterium-labeled analogues in a He buffer gas. NC_2_H_6_^+^ ions were prepared via dissociative electron ionization of a wide variety of alkyl amines and injected into the helium-filled collision cell. The most abundant collisional dissociation products were found to be CH_3_^+^, NH_4_^+^, NC_2_H_4_^+^, and NC_2_H_5_^+^. Lowering the electron energy reduced the internal energy of the incident NC_2_H_6_^+^ ions and resulted in a decrease in the signals from NH_4_^+^ and NC_2_H_4_^+^ and an increase in the signal from NC_2_H_5_^+^. This is similar to the behavior observed as the photon energy is reduced in the present photolysis study, except that we do not see significant amounts of NC_2_H_5_^+^ at any of the three wavelengths studied. Additionally, we also observe the formation of NCH_4_^+^, which Levsen and McLafferty did not observe. This is consistent with intramolecular hydrogen transfer in the primary amide bond dissociation step in DMF^+^ being a key step in NCH_4_^+^ formation; this proton transfer was not possible in Levsen and McLafferty’s study, which used a different precursor ion.

We caveat the above comparison by noting that in Levsen and McLafferty’s experiments, the collisional dissociation occurred around 10 μs after formation of the NC_2_H_6_^+^ ions as a result of the transport time of the ion beam to the collision region. Over this time scale, all of the ions will have isomerized to their most stable structure, and any that have dissociated on a faster time scale than this will have been lost from the beam, along with their fragmentation products. The products observed are therefore those formed in collisions of only the most stable isomer of NC_2_H_6_^+^ with helium. In our experiment, we extract ions from the interaction region on a much faster time scale and therefore potentially see dissociation products arising from more than one isomer.

### 3.5 Comparison with Dissociative Electron Ionization

The ToF spectrum obtained in the present work at 225 nm is similar to the 70 eV electron ionization mass spectrum reported by Li et al.^23^ A greater degree of secondary fragmentation was observed in Li’s work relative to ours, presumably due to the different internal energies of the primary NC_2_H_6_^+^ fragment ions formed via the two different ionization mechanisms. Following electron ionization, Li et al. observed straightforward N–CO or N–CH_3_ bond cleavage and also noted a channel involving N–CO bond fission and simultaneous intramolecular hydrogen transfer from the HCO carbonyl end to the nitrogen atom, followed by loss of one of the methyl hydrogen atoms to form NC_2_H_6_^+^. Interestingly, we see N–CH_3_ bond cleavage as a result of the initial 118 nm photoionization step, but we do not see further N–CH_3_ bond dissociation in the DMF^+^ cation following absorption of a UV photon. As noted earlier, absorption of a UV photon by the DMF^+^ cation appears to lead almost exclusively to cleavage of the amide bond, either with or without simultaneous intramolecular hydrogen transfer, with the resulting NC_2_H_6_^+^ ion able to decompose via a variety of pathways to yield our observed products. All of these products, apart from CH_3_^+^, were also observed and studied by Li et al. Many of these processes had also been studied previously by Loudon and Webb^24^ through analyzing metastable peak shapes within a commercial mass spectrometer, an analysis which provides insight into the initial and final masses and the conversion between them.

## 4 Conclusions

We have reported results from a comprehensive study of the UV photolysis of the DMF^+^ cation. The DMF^+^ ions are prepared by single-photon ionization of neutral DMF at 118 nm, yielding DMF^+^ parent ions accompanied by small amounts of HN(CH_2_)(CH_3_)^+^ and HCONHCH_2_^+^/OCNHCH_3_^+^ resulting from N–CO or N–CH_3_ bond cleavage, respectively. In the subsequent UV-induced photolysis of the parent ion, the N–CO amide bond breaks selectively, yielding HCO + NC_2_H_6_^+^ as major products. These products can form on all low-lying electronic states of the ion, except for the 3^2^A′ state, which produces HCO^+^ + NC_2_H_6_. Virtually all of the available energy is released into internal modes of the NC_2_H_6_^+^ ion and its coproduct(s), with only a few percent of the energy released into translation. In addition to these simple N–CO bond cleavage pathways, N–CO bond dissociation can be accompanied by simultaneous intramolecular hydrogen transfer from the oxygen side of the molecule to the nitrogen atom, in which case NCH_4_^+^ can be formed. This channel is low in energy and appears to proceed efficiently at all three photolysis wavelengths studied.

The NC_2_H_6_^+^ ion formed in the primary dissociation step has a lifetime of at least tens of microseconds^43^ and is fluxional in structure, readily isomerizing from  to H_2_C–NH–CH_3_^+^. It can dissociate further via a number of different pathways to produce CH_3_^+^, NH_4_^+^, NCH_2_^+^, and NC_2_H_4_^+^. The abundance of the various fragment ions increases with increasing photon energy, which is in line with increasing internal energy of the primary dissociation products. This is consistent with the velocity-map images, which are all isotropic and show little dependence on the photolysis photon energy over the range studied. We interpret the lack of dependence of the images on photolysis energy as indicating that dissociation occurs from the same set of electronic states at all wavelengths studied, most likely the ionic ground state (1^2^A′) and first excited state (2^2^A′).

DMF provides a useful model for a peptide bond, and the present study offers some insight into the dynamics of possible peptide fragmentation pathways, including evidence for intramolecular hydrogen atom transfer. However, in order to obtain a more complete understanding of the behavior of peptide cations following absorption of a UV photon, further studies on more complex model compounds are clearly required in order to investigate the effect of amino acid side chains, multiple peptide bonds, charge localization or delocalization over larger distances, and many other factors.

## Supporting Information Available

Optimized internal coordinates for DMF and DMF^+^, and potential energy curves for DMF^+^ (PDF)
