# Supplementary material for: Ultraviolet Photodissociation of the N,N-Dimethylformamide Cation
Source: J Phys Chem A. 2024 Dec 3;128(49):10525–33. doi: 10.1021/acs.jpca.4c06227 (PMC11647886; doi:10.1021/acs.jpca.4c06227)
Supplement: Supplementary file 1 — jp4c06227_si_001.pdf [file jp4c06227_si_001.pdf]

Supplementary information: Ultraviolet photodissociation of the *N,N*-dimethylformamide cation

Dennis Milesevic, Alexander Butler, Patrick Robertson, and Claire Vallance*
Department of Chemistry, University of Oxford,
Chemistry Research Laboratory, 12 Mansfield Rd, Oxford OX1 3TA, UK
*e-mail: claire.vallance@chem.ox.ac.uk

1 Optimised internal coordinates for DMF and DMF^+

Table 1: Internal coordinates for the DMF cation and neutral optimized in UMP2/aug-cc-pVTZ calculations. The respective atom numbers are indicated by the numbers in parentheses, and refer to the atom numbers in Figure 1 of the main manuscript.

Coordinate	DMF^+ cation	DMF neutral	Coordinate	DMF^+ cation	DMF neutral
$R(1,2)$ /Å	1.098	1.101	$A(4,9,11)$ /°	109.10	110.73
$R(1,3)$ /Å	1.148	1.223	$A(4,9,12)$ /°	108.17	110.73
$R(1,4)$ /Å	1.551	1.357	$A(10,9,11)$ /°	111.02	108.68
$R(4,5)$ /Å	1.430	1.446	$A(10,9,12)$ /°	110.09	108.68
$R(4,9)$ /Å	1.431	1.443	$A(11,9,12)$ /°	107.66	108.47
$R(5,6)$ /Å	1.100	1.091	$D(2,1,4,5)$ /°	177.18	178.00
$R(5,7)$ /Å	1.088	1.086	$D(2,1,4,9)$ /°	-0.73	-0.00
$R(5,8)$ /Å	1.086	1.091	$D(3,1,4,5)$ /°	-2.59	0.0
$R(9,10)$ /Å	1.087	1.088	$D(3,1,4,9)$ /°	179.50	180.00
$R(9,11)$ /Å	1.093	1.092	$D(1,4,5,6)$ /°	-84.52	-119.93
$R(9,12)$ /Å	1.096	1.092	$D(1,4,5,7)$ /°	31.85	-0.01
$A(2,1,3)$ /°	131.12	122.54	$D(1,4,5,8)$ /°	156.62	119.91
$A(2,1,4)$ /°	109.46	111.88	$D(9,4,5,6)$ /°	93.35	60.07
$A(3,1,4)$ /°	119.42	125.59	$D(9,4,5,7)$ /°	-150.27	179.99
$A(1,4,5)$ /°	117.06	120.45	$D(9,4,5,8)$ /°	-25.50	-60.10
$A(1,4,9)$ /°	120.75	121.78	$D(1,4,9,10)$ /°	6.45	-0.00
$A(5,4,9)$ /°	122.16	117.77	$D(1,4,9,11)$ /°	128.90	119.82
$A(4,5,6)$ /°	107.26	110.32	$D(1,4,9,12)$ /°	-114.25	-119.82
$A(4,5,7)$ /°	109.30	108.21	$D(5,4,9,10)$ /°	-171.35	180.00
$A(4,5,8)$ /°	110.05	110.32	$D(5,4,9,11)$ /°	-48.90	-60.18
$A(6,5,7)$ /°	107.60	109.62	$D(5,4,9,12)$ /°	67.95	60.18
$A(6,5,8)$ /°	109.34	108.74			
$A(7,5,8)$ /°	113.11	109.62			
$A(4,9,10)$ /°	110.70	109.50			

2 Potential energy curves for DMF$^+$

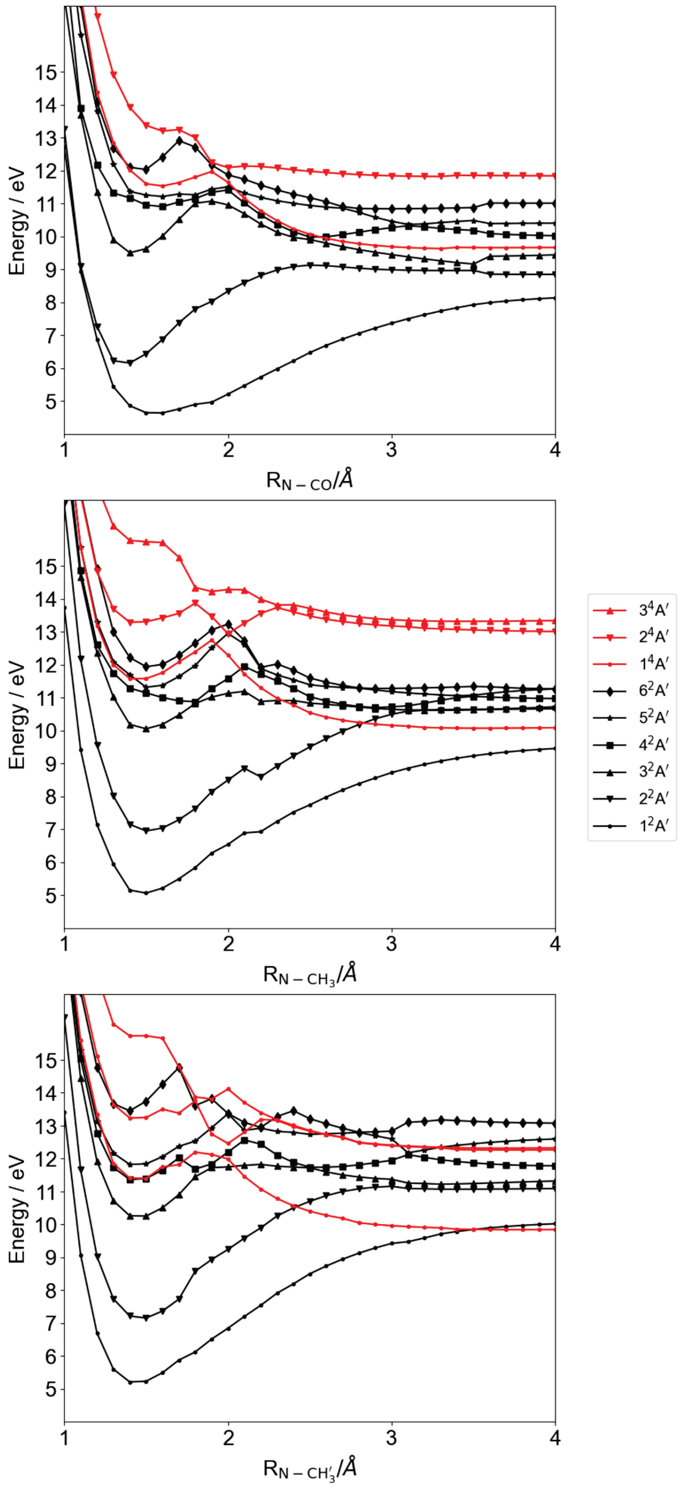

Figure 1: Potential energy curves for dissociation of the DMF$^+$ cation along the N−CO stretch coordinate (upper panel), the N−CH_3 coordinate on the side of the oxygen atom (central panel), and the remaining N−CH_3 bond (lower panel) calculated in the CASSCF calculations. See main text for details of calculations.
